# The Impact of HIV-1 Genetic Diversity on CRISPR-Cas9 Antiviral Activity and Viral Escape

**DOI:** 10.3390/v11030255

**Published:** 2019-03-13

**Authors:** Gilles Darcis, Caroline S. Binda, Bep Klaver, Elena Herrera-Carrillo, Ben Berkhout, Atze T. Das

**Affiliations:** 1 Laboratory of Experimental Virology, Department of Medical Microbiology, Amsterdam University Medical Centers, 1105AZ Amsterdam, The Netherlands; c.s.binda@amc.uva.nl (C.S.B.); g.p.klaver@amc.uva.nl (B.K.); e.herreracarrillo@amc.uva.nl (E.H.-C.); 2 Infectious Diseases Department, Liège University Hospital, 4000 Liège, Belgium; gdarcis@chuliege.be

**Keywords:** HIV-1, subtypes, isolates, diversity, CRISPR-Cas9, dual-gRNA, escape, cure

## Abstract

The clustered regularly interspaced short palindromic repeats (CRISPR)-Cas9 system is widely explored for sequence-specific attack on HIV-1 proviral DNA. We recently identified dual-guide RNA (dual-gRNA) combinations that can block HIV-1 replication permanently in infected cell cultures and prevent viral escape. Although the gRNAs were designed to target highly conserved viral sequences, their efficacy may be challenged by high genetic variation in the HIV-1 genome. We therefore evaluated the breadth of these dual-gRNA combinations against distinct HIV-1 isolates, including several subtypes. Replication of nearly all virus isolates could be prevented by at least one gRNA combination, which caused inactivation of the proviral genomes and the gradual loss of replication-competent virus over time. The dual-gRNA efficacy was not affected by most single nucleotide (nt) mismatches between gRNA and the viral target. However, 1-nt mismatches at the Cas9 cleavage site and two mismatches anywhere in the viral target sequence significantly reduced the inhibitory effect. Accordingly, sequence analysis of viruses upon breakthrough replication revealed the acquisition of escape mutations in perfectly matching and most 1-nt mismatching targets, but not in targets with a mismatch at the Cas9 cleavage site or with two mismatches. These results demonstrate that combinatorial CRISPR-Cas9 treatment can cure T cells infected by distinct HIV-1 isolates, but even minor sequence variation in conserved viral target sites can affect the efficacy of this strategy. Successful cure attempts against isolates with divergent target sequences may therefore require adaptation of the gRNAs.

## 1. Introduction

Combination antiretroviral therapy (cART) increases survival and quality of life of HIV-1 infected patients. However, cART is not curative and HIV-1 persists in viral reservoirs [1]. These reservoirs, defined as a cell type or anatomical site where a replication-competent form of the virus persists during therapy, are established during the first days of infection [2,3,4]. Although the reservoirs are heterogeneous in nature, the main reservoir is thought to reside in latently-infected CD4^+^ memory T cells [5,6,7]. The integrated proviral DNA in the reservoir cells can give rise to infectious virus production upon activation by various stimuli, leading to viral rebound when cART is interrupted [1,4,8,9]. Therapeutic strategies that eliminate these proviruses in the reservoir cells may lead to a cure.

In the past decade, several DNA editing tools, including tailored recombinases, zinc finger nucleases (ZFN), transcription activator-like effector nucleases (TALEN) and homing endonucleases, have been developed that open the way to such novel antiviral strategies [10,11,12,13,14,15,16]. Due to its very high specificity and flexibility, the bacterial clustered regularly interspaced short palindromic repeats (CRISPR)-Cas9 system is now widely used, not only for genome editing but also in antiviral applications [17,18,19,20,21]. Sequence specificity of this CRISPR-Cas9 system is mediated by 20 nucleotides (nt) at the 5′ end of the guide RNA (gRNA) that direct Cas9 to a complementary sequence (the protospacer) in the target DNA. Only complementary sequences located immediately upstream of a short nt motif, the protospacer adjacent motif (PAM; NGG for *Streptococcus pyogenes* Cas9), will be cleaved by Cas9.

Several studies investigated whether the CRISPR-Cas9 system can be used to cleave and inactivate the HIV-1 provirus in infected cells (reviewed in [19]). Combining Cas9 with a single gRNA targeting HIV-1 DNA can indeed inhibit viral gene expression and replication in infected cell cultures. However, the virus frequently escapes from this inhibition by acquisition of mutations in the Cas9 cleavage site, which provides resistance against Cas9 attack [22,23,24]. Whereas HIV-1 usually evolves via mutations introduced during error-prone reverse transcription, the mutations at the Cas9 cleavage site are generated through the non-homologous end-joining (NHEJ) DNA repair process that is activated upon Cas9-mediated DNA cleavage [22,24]. Rapid escape was observed when non-essential viral sequences were targeted, as these sites can accommodate the typical indel mutations that are introduced during NHEJ repair. In contrast, virus escape was delayed when essential protein-encoding HIV sequences were targeted, as these highly conserved sequences do not easily tolerate indels. Point mutations were selected at these sites, but such mutations occur less frequently during DNA repair or virus replication, which explains the delay in viral escape [22]. Combining two gRNAs targeting different HIV-1 domains can further delay viral escape, but escape was nevertheless observed for most dual-gRNA combinations due to NHEJ-introduced mutations in both viral targets [25,26]. By testing multiple gRNA combinations, we identified two dual-gRNA sets that completely blocked virus replication in infected cell cultures [25]. In these protected cells, continuous Cas9 attack generated hypermutated proviruses, which coincided with the loss of replication-competent virus.

We thus provided the proof of principle that the CRISPR-Cas9 system can be used to cure HIV-1 infected cells in an in-vitro cell culture system [25]. However, these studies were performed with a single HIV-1 subtype B strain. Whether the dual-gRNA combinations can inhibit and permanently inactivate other virus isolates should therefore be investigated. This question is of the utmost importance since one of the characteristics of the worldwide HIV-1 spread is the high genetic variation among virus isolates. HIV-1 strains have been classified according to their phylogenetic relationship [27]. HIV-1 group M is the most widespread and is subdivided into nine subtypes designated by the letters A–D, F–H, J and H [28]. Co-infection with multiple strains or subtypes led to the generation of new circulating recombinant forms (CRFs) or unique recombinant forms (URFs). Several genome-editing studies in which the DNA targeting specificity of Cas9 was analyzed, demonstrated that nucleotide mismatches between the gRNA and DNA target can reduce the Cas9 cleavage activity, in particular mismatches in the PAM-proximal region [29,30,31]. Furthermore, the absence of a proper PAM motif will preclude Cas9 activity. Thus, the considerable genetic variation of HIV-1 may compromise the CRISPR-Cas9 based therapy [32,33]. We therefore evaluated the breadth of the combinatorial approach by testing the capacity of the best dual-gRNA combinations to inhibit and inactivate distinct HIV-1 isolates, including non-B subtypes.

## 2. Materials and Methods

### 2.1. Plasmids

The lentiviral vector pLenti-SpBsmBI-gRNA-Hygro (Addgene; 62205) used for the expression of gGag1 was a gift from Rene Maehr [34]. The lentiviral vector LentiGuide-Puro (Addgene; 52963) used for the expression of the other gRNAs (Env2, TatRev and Luc) and LentiCas9-Blast (Addgene; 52962) containing the human codon-optimized *Streptococcus pyogenes* Cas9-expression cassette were gifts from Feng Zhang [35]. Ligation of the gRNA-encoding oligonucleotides into the pLenti-SpBsmBI-sgRNA-Hygro and LentiGuide-Puro vectors was described previously [22,25].

### 2.2. Cell Culture, Transfection and Transduction

Human embryonic kidney 293T cells and Jurkat T-cells were cultured as described previously [36,37]. Lentiviral vectors were produced as previously described [38]. Briefly, the vectors were produced by transfection of 293T cells with the lentiviral vector plasmid and packaging plasmids pSYNGP, pRSV-rev, and pVSV-g using Lipofectamine 2000. After transfection, the medium was replaced with Opti-MEM (Life Technologies), and the cells were cultured for 48 h. The lentiviral vector containing supernatant was centrifuged and filtered (0.45 μm). The LentiCas9-Blast vector particles were concentrated using a Vivaspin 20 ultrafiltration spin column (100 kDa molecular weight cutoff; Sartorius). Vector aliquots were stored at −80 °C. Virus particle production was measured by CA-p24 ELISA as previously described [39]. Jurkat cells (2 × 10^5^ cells in 1 mL of culture medium) were transduced with LentiCas9-Blast virus particles (30 ng of CA-p24) and cultured with 3 μg/mL blasticidin for 10 days to select Jurkat-Cas9 cells. These cells were subsequently transduced with pLenti-SpBsmBI-gGag1-Hygro or LentiGuide-Puro (gEnv2, gTatRev or gLuc) virus particles and cultured with 500 μg/mL hygromycin B or 1 μg/mL puromycin, respectively, for 2 weeks to select cells expressing Cas9 and a single gRNA. To produce Jurkat cells expressing Cas9 and two gRNAs, the Jurkat-Cas9 cells were first infected with the pLenti-SpBsmBI-gGag1-Hygro virus followed by hygromycin B selection and subsequently with the LentiGuide-Puro-gEnv2 or gTatRev virus followed by puromycin selection.

### 2.3. Virus Stocks and Infection

**Figure 1:**
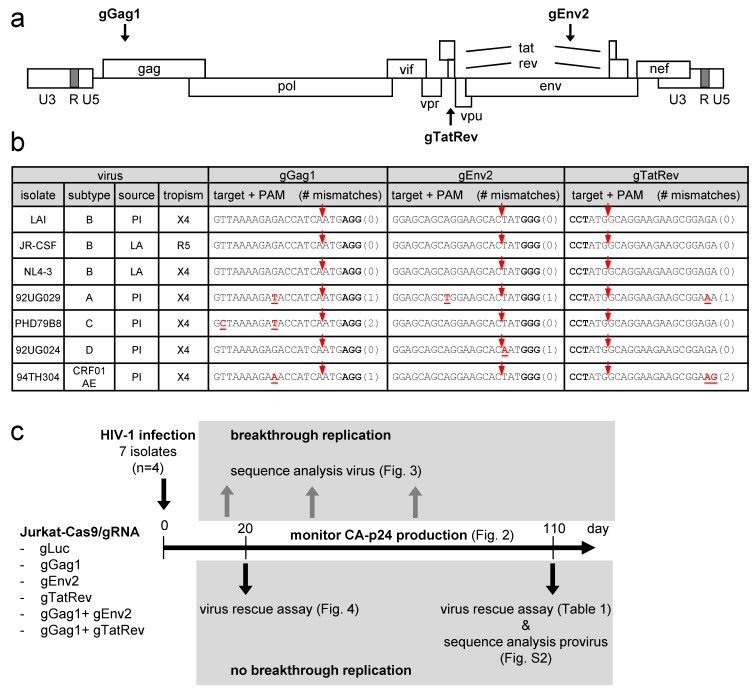
CRISPR-Cas9 targeting of different HIV-1 isolates. (**a**) The HIV-1 proviral genome with the position of the gRNA-target sites indicated. The gRNAs binding to the sense and antisense DNA strand are indicated above and below the proviral genome, respectively. (**b**) Different HIV-1 isolates tested in this study. For each isolate, the subtype, source (PI, primary isolate; LA, laboratory-adapted) and tropism (X4, CXCR4-using; R5, CCR5-using) are indicated. The gRNA-target sequence is shown with nucleotides mismatching with the gRNA in red and underlined (number of mismatching nt indicated between brackets) and the PAM nucleotides in bold. The Cas9 cleavage site is indicated with a red arrow. (**c**) Experimental design. See text for details.

The viruses listed in Figure 1b were obtained from the NIH AIDS Research and Reference Program (Division of AIDS, NIAID, NIH) or isolated from HIV-infected patients in Ethiopia and the Netherlands [40]. Virus stocks were generated and titrated as described previously [39,41]. Cas9 and gRNA-expressing Jurkat cells (2 × 10^5^ cells in 1 mL of culture medium) were challenged with each HIV-1 isolate at a MOI of 0.001. Cells were passaged twice a week and virus production was monitored by CA-p24 ELISA of culture supernatant samples, as previously described [42].

### 2.4. Sequencing Analysis and Virus Rescue Assay

To analyze the gRNA-targeted proviral sequence in infected dual-gRNA-expressing cell cultures demonstrating virus replication, cell-free virus-containing supernatant was isolated at the peak of infection and passaged to fresh matching dual-gRNA-expressing cells. When virus replication was detected in these cells, cellular DNA with the integrated provirus was isolated as described previously [25]. The gRNA target region in the proviral DNA was PCR amplified and sequenced with primer pairs listed in Table S1. To analyze the proviral gRNA-target region in cultures that did not demonstrate breakthrough virus replication, infected cells were harvested by centrifugation and the cellular DNA with the integrated provirus was isolated with the DNAeasy kit (Qiagen). The gRNA target region was amplified by PCR (primers listed in Table S1). The PCR product was cloned in a TA cloning vector and the sequence of cloned fragments was analyzed. To demonstrate the presence or absence of replication-competent proviruses in these non-breakthrough cultures, the infected cells were mixed with an equal amount of control (non-transduced) Jurkat cells and virus production was monitored by CA-p24 ELISA during co-culturing of the cells for 21 days.

## 3. Results

### 3.1. Experimental Design for Evaluating the Breadth of the CRISPR-Cas9 Dual-gRNA Therapy against Diverse HIV-1 Isolates

We previously described the gGag1, gEnv2 and gTatRev gRNAs that target gag, env and overlapping tat and rev sequences in the HIV-1 proviral DNA, respectively (Figure 1a) [22]. Although these individual gRNAs potently reduced viral gene expression when co-expressed with Cas9, virus replication was only transiently inhibited, which was due to the acquisition of escape mutations in the gRNA target site [22]. Viral escape was not observed when gGag1 was combined with gEnv2 or gTatRev and these dual-gRNA combinations durably inhibited virus replication [25]. The 20-nucleotide spacer of these gRNAs, which defines the DNA target site, was based on the HIV-1 LAI isolate, a subtype B virus that uses the CXCR4 co-receptor for viral entry. Although the gRNAs were designed to target highly conserved sequences, a recent *in silico* analysis predicted that they would be effective against 82 to 95% of the HIV-1 subtype B variants described in the Los Alamos National Library HIV sequence database [43]. To evaluate the breadth of these dual-gRNA CRISPR-Cas9 therapies, we set out to test their capacity to inhibit replication of diverse HIV-1 isolates, including viruses corresponding to other subtypes (A, C, D and CRF01_AE; listed in Figure 1b), which were expected to show sequence variation in the gRNA targets. Both primary isolates (PI) and laboratory-adapted (LA) strains using either CXCR4 (X4) or CCR5 (R5) as co-receptor for viral entry were tested.

SupT1 T cells, which we routinely use to study HIV-1 replication, do not express R5. For this reason, Jurkat T-cells, which express both X4 and R5 and support replication of all HIV-1 isolates, were used in this study. The Jurkat cells were transduced with Cas9 and gRNA-expressing lentiviral vectors. We thus generated gGag1, gEnv2 and gTatRev single-gRNA/Cas9 cells and gGag1 + gEnv2 and gGag1 + gTatRev dual-gRNA/Cas9 cells. As a control, we also produced cells expressing Cas9 and a control gRNA that targets the firefly luciferase gene (gLuc). The experimental design is shown in Figure 1c. The Jurkat-Cas9-gRNA cell lines were infected with the different viruses, with every virus-cell combination being tested in 4 cultures (*n* = 4). Virus replication was subsequently monitored for up to 110 days by measuring the viral capsid protein (CA-p24) in the culture medium and checking for cytopathic effects (CPE) twice a week. When the CA-p24 level increased strongly, reflecting breakthrough virus replication, the sequence of the gRNA target region in the viral genome was analyzed to check for escape mutations. Furthermore, for cultures that did not show any sign of virus replication up to day 110 (no detectable CA-p24 and CPE), we tested for the presence of replication-competent viral genomes in a sensitive virus rescue assay and we sequenced the integrated proviral DNA to check for inactivating mutations.

### 3.2. Impact of Sequence Variation in the gRNA Target Region on the Efficacy of Single gRNAs

As the gRNA-target sequence was not available for all HIV-1 isolates, we first determined the corresponding gag, env and tat/rev sequences. For this, wild-type Jurkat cells were infected with the virus stocks, followed by sequence analysis of the integrated proviral DNA. As shown in Figure 1b, the gRNA-target sequences, i.e. the 20-nt protospacer and 3-nt PAM sequences, are indeed well-conserved. Notably, the target sites in all 3 subtype B isolates are identical and matched perfectly with the gRNAs. The other subtypes differ at most at 1 or 2 nt positions for some targets. The nucleotides mismatching with the gRNA and the number of mismatches (0, 1 or 2) are indicated for each isolate and target in Figure 1b. The A isolate has a 1-nt mismatch in all 3 targets, the C isolate mismatches at 2 positions in the gGag1 target only, and the D isolate mismatches at a single position in the gEnv2 target. The CRF01_AE recombinant (AE) carried a 1-nt mismatch in the gGag1 site and a 2-nt mismatch in the gTatRev site. These mismatches in the gRNA-target DNA duplex may reduce Cas9 cleavage activity. Some are located close to the Cas9 cleavage site (at 3 nt from the PAM) whereas others are located more distally, which may differentially affect Cas9/gRNA activity.

**Figure 2:**
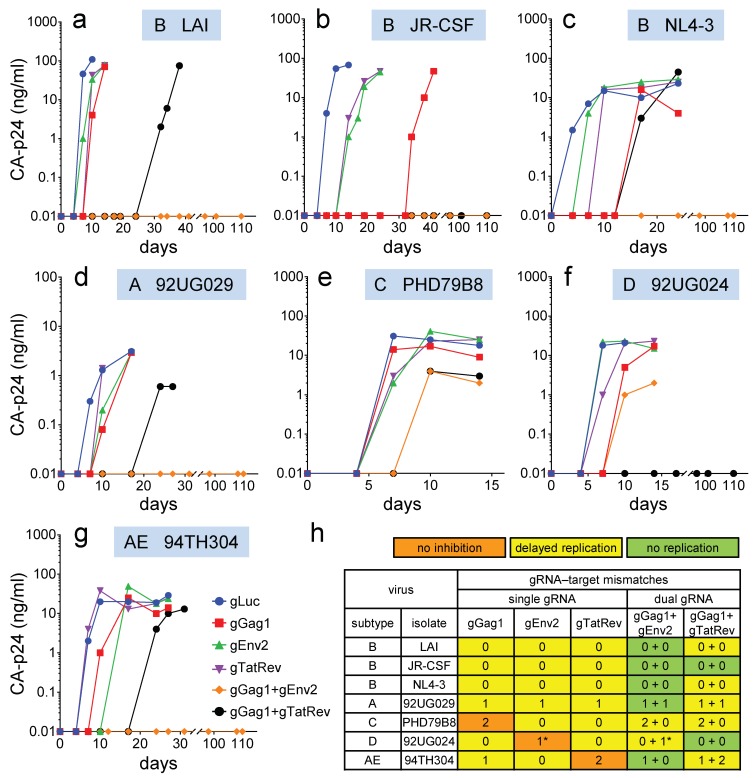
Replication of HIV-1 isolates in Cas9-gRNA-expressing T cells. Jurkat T-cells stably transduced with lentiviral vectors expressing Cas9 and single or dual gRNAs were infected with the different HIV-1 isolates and cultured for up to 110 days. (**a–g**) The CA-p24 level in the culture supernatant was measured to monitor virus replication. All viruses were tested in four cultures and one representative culture is shown. (**h**) Replication in the presence of the antiviral gRNAs was compared to replication in control gLuc-expressing cells (as shown in a–g). The color indicates the effect of the gRNAs on virus replication (orange, no inhibition; yellow, delayed replication; green, no replication). The number of nt mismatches in the viral targets are shown (*, nt mismatch at Cas9 cleavage site).

All HIV-1 isolates demonstrated efficient virus replication when control gLuc-expressing cells were infected, which resulted in cell death and a rapid increase in the CA-p24 level within one week after infection (representative replication curves are shown in Figure 2a–g). Upon infection of cells expressing either gGag1, gEnv2 or gTatRev, most isolates demonstrated a delay in the increase in CA-p24 and CPE, indicating inhibition of virus replication (data summarized in Figure 2h). Although the capacity of the gRNAs to delay virus replication varied for the different isolates, breakthrough replication of all viruses was observed within several weeks.

Most notably, no virus inhibition was scored for isolates with two mismatches in the target sequence (C isolate in gGag1 cells and AE isolate in gTatRev cells; orange in Figure 2h). This result indicates that Cas9 does not tolerate two mismatches in the target sequence, not even when these nt are positioned far from the PAM, like in the AE isolate. Replication of virus isolates without mismatches was consistently delayed (yellow in Figure 2h), irrespective of the subtype, receptor tropism (R5 or X4) or source (primary isolate or laboratory-adapted strain). Similarly, replication of virus isolates with a 1-nt mismatch in the middle of the target sequence or more distal to the PAM was transiently inhibited (A isolate in gGag1, gEnv2 and gTatRev cells; AE isolate in gGag1 cells). In contrast, replication of the D isolate was not noticeably inhibited by gEnv2, while this virus also carries only a single mismatching nt in the target sequence. However, this mismatching nt is positioned at 3 nt from the PAM sequence, immediately adjacent to the Cas9 cleavage site (between nt positions 3 and 4). This position is likely responsible for the resistance to Cas9 activity. Indeed, it has been demonstrated that Cas9 tolerates mismatches in the PAM-distal region better than in the PAM-proximal (Seed) region [30,31,44].

### 3.3. Combination of Effective gRNAs Can Durably Block Virus Replication

We will first discuss replication of the HIV-1 isolates in cells simultaneous expressing gGag1 and gEnv2. This combination completely blocked replication of the B, A and AE isolates up to 110 days (green in Figure 2h). When applied as single gRNA, both gGag1 and gEnv2 delayed but did not prevent eventual virus replication, indicating a synergistic effect when combined. In contrast, replication of the C isolate was only delayed in these dual-gRNA cells. This can be explained by the fact that only gEnv2 delayed replication of this virus to a noticeable extent, whereas gGag1 was less effective due to two mismatches in the target. The combination was more effective than gEnv2-only, indicating that gGag1 contributed somewhat to the observed inhibition. Replication of the D isolate in the gGag1 + gEnv2 cells was delayed to a similar extent as in the gGag1-only cells, which is in agreement with the ineffectiveness of gEnv2 against this virus due to a mismatch in the cleavage site.

In cells expressing both gGag1 and gTatRev, replication of the B isolate JR-CSF and the D isolate was durably blocked. This dual gRNA set delayed replication of the other isolates to a larger extent than the individual gRNAs. These results confirm the synergistic effect of gRNA combinations. For gRNAs with two mismatches that did not noticeably delay replication when applied individually (gGag1 targeting of C and gTatRev targeting of AE isolates), a contribution was observed to dual gRNA inhibition (cf. gTatRev and gGag1+gTatRev effect on C and gGag1 and gGag1+gTatRev effect on AE in Figure 2e and Figure 2g, respectively).

In conclusion, we identified at least one dual-gRNA/Cas9 treatment that completely blocked virus replication of 6 out of 7 tested isolates. Both dual-gRNA strategies could inhibit but did not prevent replication of the C isolate with two mismatches in the gGag1 target. This gRNA was part of both combinations.

### 3.4. Virus Escape in Dual-gRNA Protected Cells

**Figure 3:**
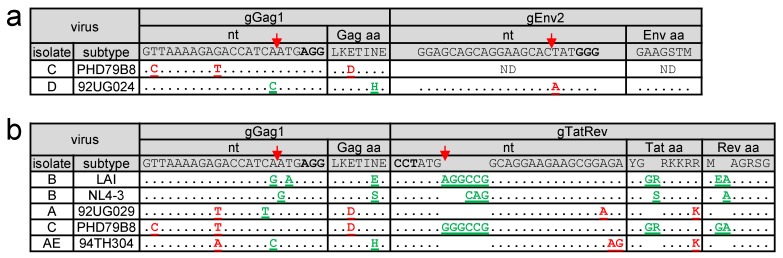
Sequence of the target region upon breakthrough virus replication. (**a–b**) When virus replication was observed in dual-gRNA expressing Jurkat cells (gGag1 + gEnv2 cells in a; gGag1+gTatRev cells in b), the viral target sequence was analyzed. The sequence fully complementary to the gRNA and the PAM (in bold), as present in the LAI isolate, are shown on top. The Cas9 cleavage site is indicated with a red arrow. For each isolate, the target sequence of the breakthrough virus is shown, with pre-existing gRNA-mismatching nucleotides indicated in red and newly acquired mutations observed upon replication indicated in green. The amino acid sequence of the encoded proteins is shown with the pre-existing and acquired amino acid differences indicated in red and green, respectively.

In previous studies in which HIV-1 was inhibited by gRNAs that are fully complementary to the viral DNA, breakthrough virus replication was linked to the acquisition of escape mutations in the gRNA target sequence. However, when the sequence of the virus isolate is not fully complementary to the gRNA, Cas9 cleavage is possibly less effective and breakthrough replication may not require additional target-site mutations. We therefore inspected the gRNA-target sequence when virus replication was observed in dual-gRNA expressing cells. Virus isolated at the peak of replication was passaged to fresh, matching dual-gRNA cells and the integrated proviral DNA produced in these infected cells was analyzed when CA-p24 was detectable in the culture medium. In Figure 3, the target sequence of the input virus and the replicating virus are shown, with gRNA-mismatching nucleotides in the input virus indicated in red and mutations observed upon replication indicated in green. In addition, the effects on the amino acid sequence of the encoded proteins are shown.

If the input viral sequence fully matched with the gRNA, this analysis consistently revealed newly acquired mutations in the target sequence (gGag1 + gEnv2 and gGag1+gTatRev results shown in Figure 3a and Figure 3b, respectively). These escape mutations clustered around the Cas9 cleavage site at 3 nt from the PAM, which indicates that these mutations are introduced during the NHEJ DNA repair that follows Cas9-mediated DNA cleavage [22]. We exclusively observed substitutions and 3-nt insertions that do not abrogate the open reading frame, which is consistent with previous findings [22]. Also, if the viral target sequence mismatched only at a single nt position, most viruses acquired additional NHEJ-induced escape mutations.

When both target sites had no or only a single mismatch, both target sites were found to be mutated in the breakthrough virus, except for the gGag1 + gEnv2 targeted D isolate and the gGag1 + gTatRev targeted A isolate. The D isolate did acquire a 1-nt substitution in the fully matching gGag1 site, but no mutations were observed in the 1-nt mismatching gEnv2 site, which can be explained by the position of this mismatch at the Cas9 cleavage site. The A isolate did acquire a mutation in the gGag1 but not the gTatRev target, while both have a 1-nt mismatch, located at a central and more PAM-distal position, respectively. When applied as single gRNAs, gTatRev did inhibit replication of the A isolate less effectively than gGag1 (Figure 2). These combined results suggests that some pre-existing target mismatches can reduce the gRNA/Cas9 activity to such an extent that no further mutations are required to obtain breakthrough virus replication.

The C isolate with two mismatches in the gGag1 site and no mismatches in the gTatRev site acquired additional mutations in the gTatRev site and not the gGag1 site upon replication in gGag1+gTatRev cells. Similarly, the AE isolate acquired escape mutations in the 1-nt mismatching gGag1 site but not in the 2-nt mismatching gTatRev site upon replication in gGag1+gTatRev cells. These results are in line with the poor inhibition of the C and AE isolates in gGag1-only and gTatRev-only cells, respectively.

Taken together, these results demonstrate that when the viral sequence is fully complementary to the gRNAs, the virus can only overcome the gRNA/Cas9 mediated inhibition by acquisition of escape mutations in the target sequences. For most viruses with single nt mismatches, we observed the same pattern of inhibition and eventual escape. However, virus inhibition is reduced by some 1-nt mismatches, e.g. a mismatch at the Cas9 cleavage site, and by 2-nt mismatches in the target site, thereby avoiding the need to acquire additional mutations.

### 3.5. Complete Virus Inactivation in Dual-gRNA Protected Cells

We checked for the presence of replication-competent virus in dual-gRNA cell cultures that did not show any sign of virus replication (CA-p24, CPE) at 20 and 110 days after infection. For this, a virus rescue assay was used in which infected cells were mixed with wild-type, unprotected Jurkat cells. These cell mixtures were subsequently cultured for 21 days to monitor the spread of replication-competent virus by CA-p24 ELISA. For each virus, we analyzed all four gGag1 + gEnv2 and gGag1 + gTatRev cultures. At day 20, virus could be rescued from the gGag1 + gTatRev cultures infected with the B isolates LAI and NL4-3, and the A and AE isolates (Table 1). These cultures were not tested at day 110 because we observed prior breakthrough virus replication (Figure 2). For the subtype B JR-CSF infected gGag1 + gTatRev cultures and all tested gGag1 + gEnv2 cultures, the virus could not be rescued from any of the 4 cultures at day 20 or rescue occurred in a single culture. No replication-competent virus could be rescued from any of these cultures at day 110, indicating that all viral genomes were progressively and completely inactivated. Sequencing of the integrated proviral DNA did confirm that the gRNA targets were heavily mutated and no wild-type viral genomes were detected (Supplementary Figure S1). As previously observed [25], most proviral genomes acquired short indels that cluster around the Cas9 cleavage site and affect the encoded protein. Moreover, the proviral DNA was analyzed by PCR using primer sets that detect truncated proviruses resulting from cleavage at the two targets and subsequent ligation with loss of the intervening fragment. In agreement with our previous observations [25], we could also detect DNA fragments with the anticipated size (data not shown). These truncated HIV-1 genomes miss the complete Pol gene and additional essential sequences and do not encode replication competent viruses. Thus, the infected cell cultures were sterilized through Cas9/NHEJ-mediated mutation and excision of proviral DNA [25].

These results demonstrate that gGag1 + gEnv2 dual-gRNA/Cas9 treatment did cure the cells from infection with the B, A and AE isolates, whereas the gGag1 + gTatRev combination cured cells from infection with the B isolate JR-CSF and the D isolate. Thus, whereas both gRNA combinations can permanently inactivate the B isolate JR-CSF, at least one combination was able to inactivate the other viruses. Only the C isolate was not blocked by either strategy, which is likely due to the two mismatches in the target site for gGag1 that is part of both dual-gRNA sets.

## 4. Discussion

To evaluate whether HIV-1 genetic variation may compromise a CRISPR-Cas9-based cure attempt, we tested two potent dual-gRNA combinations for their capacity to inhibit the replication of diverse HIV-1 variants, including subtype A, B, C, D and AE isolates. Both gRNA sets were previously shown to be able to sterilize SupT1 T-cell cultures infected with the subtype B LAI isolate that has matching target sites. The target sites in the newly tested isolates were either fully complementary to the gRNAs or 1 or two mismatches were present. We observed that neither dual-gRNA/Cas9 strategy prevented replication of all tested isolates. However, replication of nearly all isolates could be permanently blocked by at least one of the dual-gRNA combinations. When viral replication was blocked till the end of the experiment (110 days), no replication-competent virus could be rescued from the infected cell cultures. This result indicates that all integrated proviruses had been inactivated, which was confirmed to be due to Cas9/NHEJ-mediated mutation (mostly indels in the gRNA targets) and excision of the proviral DNA.

Although our results demonstrate that combinatorial CRISPR-Cas9 treatment can cure T cells infected by distinct HIV-1 isolates, we also show that the efficacy of this therapy is compromised by sequence variation of the target sites. Evaluation of the gRNA targets in viruses that can be blocked versus those that cannot, did shed some light on the sequence criteria for a dual-gRNA strategy. We observed that 2 gRNA-target mismatches significantly reduced the antiviral effect. When tested individually, the gRNAs with two mismatches did not or not noticeably inhibit virus replication, but when combined with a second gRNA, they did improve the level of inhibition, indicating some gRNA/Cas9 activity. Most 1-nt mismatches did not significantly affect gRNA/Cas9 inhibition, but the gRNA was inactive if the mismatch was positioned at the Cas9 cleavage site.

For the mismatching gRNA-target combinations we calculated the cutting frequency determination (CFD) score and the relative cleavage efficiency (RCE) of the gRNA (Table S2). These values are based on the large-scale analysis of the Cas9 activity with single-nt mismatching gRNAs by Doench et al. [29] and Hsu et al. [30], respectively. The calculated CFD values varied from 0.60 to 0.93 for the gRNAs with 1 and 2 nt mismatches, suggesting substantial Cas9/gRNA activity for all, whereas our analysis demonstrated that the gRNAs with 2-nt mismatches or with a 1-nt mismatch at the cleavage site do not noticeably inhibit virus replication when individually applied. The calculated RCE value was 0.12 for the gRNA with a mismatch at the cleavage site, which is in agreement with the poor anti-viral activity of this gRNA. However, the RCE value of the other mismatching gRNAs varied from 0.54 to 2.11, again suggesting substantial activity for all of them. Overall, the CFD and RCE values of the mismatching gRNAs did not correlate with their capacity to inhibit virus replication and are thus poor predictors of the antiviral activity. However, our analysis was limited to a small set of mismatching gRNA-virus combinations.

Inspection of the target sites when virus replication was observed in dual-gRNA cells demonstrated the acquisition of escape mutations if the viral sequence was fully complementary to the gRNAs. These mutations, mostly nt substitutions and 3- or 6-nt insertions that do not abrogate the open reading frame, are introduced during the NHEJ DNA repair that follows Cas9 cleavage and are subsequently selected because they confer resistance against gRNA/Cas9 activity, yet do not interfere with virus replication [22,23,24]. Such escape mutations were also observed for most 1-nt mismatching viral targets, which is in agreement with the efficient inhibition of these viruses. However, some 1-nt mismatches (e.g., the 1-nt mismatch in the gTatRev target of the A isolate and the 1-nt mismatch at the Cas9 cleavage site in the gEnv2 target of the D isolate) and two mismatching nts significantly reduced the gRNA/Cas9 inhibition and breakthrough replication of these viruses did not require additional target site mutations.

The gGag1 + gEnv2 combination durably blocked replication of all isolates, except for the C isolate with two mismatches in the gGag1 target and the D isolate with a 1-nt mismatch at the Cas9 cleavage site in the gEnv2 target. The low activity of gGag1 and gEnv2, respectively, likely turned the dual-gRNA strategy effectively into a single-gRNA treatment from which the virus can readily escape [22]. Nevertheless, this dual-gRNA combination seems a good candidate for pre-clinical testing and further development of a CRISPR-Cas9-based gene therapy for the treatment of HIV-1-infected individuals. Our results demonstrate that targeting of isolates with a divergent sequence may necessitate a slight adaptation of the gRNA sequence. Alternatively, a triple-gRNA strategy may be able to better tolerate mismatches in the targets. It should be noted that in the dual-gRNA setting, the presence of two mismatches did not completely abolish antiviral activity of the gRNA. Considering the heterogeneity of the reservoir cells that should be targeted in the anti-HIV gene therapy, another complicating factor could be that the dual-gRNA/Cas9 effectivity may vary in different cell types. For example, we previously observed that the gGag1 + gTatRev combination durably blocked replication of the subtype B LAI isolate in SupT1 T cells [25], while the virus was able to escape from this inhibition in Jurkat cells in this study. Differences in expression of the gRNAs and Cas9, or in the replication capacity of the virus isolate in different cell types may lead to a different outcome.

We realize that major issues need to be addressed in order to develop a safe and effective *in vivo* gene therapy. This study demonstrates that Cas9 can tolerate 1-nt imperfections in the gRNA-DNA duplex. This can be considered an advantage since it improves the breadth of the antiviral strategy. However, it likely also increases the number of potential off-target sites, which raises safety problems. For instance, there is a risk of knocking out essential or haplo-insufficient genes or inducing mutations in tumor-associated genes. These shortcomings must be overcome or ruled out by experimental validation before CRISPR-Cas9 can be used in gene therapy applications. A number of approaches have been developed to reduce CRISPR-Cas9 off-target effects [45], including many online tools for *in silico* gRNA design and prediction of off-target sites [46,47]. The gRNAs used in our study were specifically designed to have a high on/off-targeting ratio. Cas9 variants or Cas9 orthologues with higher sequence-specificity may also reduce off-targeting [47].

Successful translation to the clinic also necessitates the effective delivery of Cas9 and gRNAs to most, if not all, latently-infected cells that constitute the HIV-1 reservoir [48,49,50,51]. Although several methods are available for transient delivery of these components, viral vector delivery methods, like the adeno-associated virus (AAV) and HIV-based lentiviral vector (LV) systems, may provide more sustained therapeutic activity [20,52,53]. A significant constraint in viral delivery is the size of the Cas9 gene, since viral vectors have restricted capacity to store genetic information. The use of smaller Cas9 variants from other bacterial species (e.g., *Staphylococcus aureus* Cas9), truncated Cas9 proteins lacking non-essential domains or small gRNA/Cas9 cassettes may reduce this problem [54,55,56,57]. The Cpf1 (Cas12a) system could be an interesting alternative, as the Cpf1 gene is relatively small and this system was reported to have increased specificity, which could mitigate both the delivery and off-target problems [58,59].

## Figures and Tables

**Table 1:** Virus rescue assay.

Virus		Cultures with Replication-Competent Virus ^1^			
		gGag1 + gEnv2		gGag1 + gTatRev	
Subtype	Isolate	day 20	day 110	day 20	day 110
B	LAI	1/4	0/4	4/4	NT
B	JR-CSF	0/4	0/4	1/4	0/4
B	NL4-3	1/4	0/4	4/4	NT
A	92UG029	1/4	0/4	4/4	NT
C	PHD79B8	NT	NT	NT	NT
D	92UG024	NT	NT	0/4	0/4
AE	94TH304	0/4	0/4	4/4	NT

^1^ Infected dual-gRNA cell cultures that did not (or not yet) demonstrate virus replication were mixed with an equal amount of wild-type, unprotected cells at 20 and 110 days after infection. Replication in these mixed cell cultures was monitored by CA-p24 ELISA to detect rescue of replication-competent virus. For each virus, all four infected cell cultures were tested and the fraction of cultures that scored positive, i.e. in which CA-p24 was detectable within the 21-day follow-up period, is indicated. NT: not tested because of breakthrough virus replication at the indicated time.

## Supplementary Materials

The following are available online at https://www.mdpi.com/1999-4915/11/3/255/s1, Figure S1: Sequence analysis of proviral DNA in dual-gRNA protected Jurkat cells, Table S1: Primers used for sequencing of gRNA target regions. Table S2: Mismatches between the gRNAs and viral target sequences.
