# Supplementary material for: The Impact of HIV-1 Genetic Diversity on CRISPR-Cas9 Antiviral Activity and Viral Escape
Source: Viruses. 2019 Mar 13;11(3):255. doi: 10.3390/v11030255 (PMC6466431; doi:10.3390/v11030255)
Supplement: Supplementary file 1 [file viruses-11-00255-s001.pdf]

viruses

MDPI

B LAI

target	mutation
GGAGCAGC AGGAAGCA C TAT**GGG**	wt
GGAGCAGC AGGAC--- C TAT**GGG**	NS/-3
GGAGCAGC AGGAAGCA CGTT TAT**GGG**	+3
GGAGCAGC AGGAAGCAAC TAT**GGG**	+1
GGAGCAGC AGGAAGCA CCCC TAT**GGG**	+3
GGAGCAGC AGGAGG-- - ---**GGG**	NS/-6
GGAGCAGC AGGAAGCA CCT TAT**GGG**	+2
GGAGCAGC AGGGAGCA CGTGACTAT**GGG**	+5

B JR-CSF

target	mutation
GGAGCAGC AGGAAGCAC TAT**GGG**	wt
GGAGCAGC AGGAC---C TAT**GGG**	NS/-3
GGAGCAGC AGGAAGCACCC TAT**GGG**	+2
GGAGCAGC --------- ------	-21
GGAGCAGC AGGAAGCACCAC TAT**GGG**	+3
GGAGCAGC AGGAAGCA- ------	-7
GGAGCAGC AGGAAGCACCCCACCTAT**GGG**	+6
GGAGCAGC AGGAAGCACTCC TAT**GGG**	+3
GGAGCAGT GGGAATAGGAGCTTTTAT**G**TT	NS/+6
GGAGCAGC AGGAAGCAC ------	-12
GGAGCATCCAGGAAGCACT TAT**GGG**	NS/+2

B NL4-3

target	mutation
GGAGCAGCAGGAAGCAC TAT**GGG**	wt
GGAGCAGCAGGAAGCACGCGTT TAT**GGG**	+5
GGAGCAGCAGGAAGCACTC TAT**GGG**	+2
GGAGCAGCAGGAAGCACTT TAT**GGG**	+2
GGAGCAGCAGGAAGCACCT TAT**GGG**	+2
GGAGCAGCAGGAAGCACTTA TAT**GGG**	+3
GGAGCAGCAGGAAGCACGGG TAT**GGG**	+3
GGAGCAGCAGGAAGCACTCC TAT**GGG**	+3
GGAGCAGCAGGAAGCACGGGCAA TAT**GGG**	+6
GGAGCAGCAGGAAGCACCCT TAT**GGG**	+3
GGAGCAGCAGGAAGCACCT TAT**GGG**	+2
GGAGCAGCAGGAAGCACTTC TAT**GGG**	+3
GGAGCAGCAGGAAGCGGTACCCTCT TAT**GGG**	NS/+8
GGAGCAGCAGGAAGCACTTGTTGAAGTGGGATTAT**GGG**	+15
GGAGCAGCT-------- ------	-20

A 92UG029

target	mutation
GGAGCAGCTGGAAGCAC TAT**GGG**	wt
GGAGCAGTCGGT----- --T**GGG**	NS/-7
GGAGCCCCGCAA----- ------	NS/-11
GGAGCATCCCGGGGAGCT TAT**GGG**	NS/+1
GGAGCAGCTGGAAGCACTTCTAACCTTAT**GGG**	+9

AE 94TH304

target	mutation
GGA GCAGCAGGAAGCAC TAT**GGG**	wt
GGA GCAGCAGGAAGCACCCCCCTTCCTAT**GGG**	+9
GGT GCAGCAGGAAGCACCAA TAT**GGG**	+3
GCTCGCCGCAAGAAGCACA TAT**GGG**	NS/+2
GGG GC------------ TAT**GGG**	-12
GCA GCAGCAGGCGGT-C TAT**GGG**	NS/-1
GGA GCAGTCGTGGGGAC TA----	NS/-12
GGGCGCA----------- ------	NS/-24

Figure S1. Sequence analysis of HIV-1 DNA in dual-gRNA protected Jurkat cells. Cellular DNA was isolated from the infected gGag1+gEnv2 cell cultures that did not show any sign of virus replication at 110 days after infection. The gEnv2-target region of the integrated proviral DNA was amplified by PCR and TA cloned. Multiple TA clones were sequenced (LAI, 7 clones; JR-CSF, 10 clones; NL4-3, 14 clones; 92UG029, 4 clones; 94TH304, 7 clones). Sequences were aligned to the wild-type viral sequence (wt reference sequence shown on top with the protein codon triplets indicated with grey boxes). The PAM sequence is indicated in bold. Mutations are shown in red (-x/+x, x nt deleted/inserted; NS, non-silent amino acid substitution).

viruses

MDPI

Table S1. Primers used for sequencing of gRNA target regions

virus			target region		
sub-type	isolate	strand	gGag1	gEnv2	gTatRev
B	LAI	sense	TAAACACAGTGGGGGGACATCAAG	GCACCCACCAAGGCAAAGAGAAGAGTGG	ATATCAAGCAGGACATAACAAGG
		antisense	AATCTGGGTTCGCATTTTGGACCA	CAACCCCAAATCCCCAGGAGCTGTTGATCC	CTATGATTACTATGGACCACACA
B	JRCSF	sense	TAAACACAGTGGGGGGACATCAAG	GTGGCACTGAAGGAAATGAC	AATGGAGCCAGTAGATCCTAGC
		antisense	AATCTGGGTTCGCATTTTGGACCA	ATGCTGTTGCGCCTCAATAG	CTTCACTCTCATTGCCACTGTC
		sense[a]		GCACCCACCAAGGCAAAGAGAAGAGTGG	
		antisense[a]		CAACCCCAAATCCCCAGGAGCTGTTGATCC	
B	NL4-3	sense	TAAACACAGTGGGGGGACATCAAG	GCACCCACCAAGGCAAAGAGAAGAGTGG	AATGGAGCCAGTAGATCCTAGC
		antisense	AATCTGGGTTCGCATTTTGGACCA	CAACCCCAAATCCCCAGGAGCTGTTGATCC	CTTCACTCTCATTGCCACTGTC
A	92UG029	sense	GCCAAAATTACCCTATAGTGCAAA	GCACCCACCAAGGCAAAGAGAAGAGTGG	TATGGGGATACTTGGGAAGGA
		antisense	ACAGGGCTATACATTCTTACTA	CAACCCCAAATCCCCAGGAGCTGTTGATCC	TAGTCCATACAACTATTGCTA
C	PHD79B8	sense	TAAACACAGTGGGGGGACATCAAG	GCACCCACCAAGGCAAAGAGAAGAGTGG	CATACAATCAATGGACACTAG
		antisense	AATCTGGGTTCGCATTTTGGACCA	CAACCCCAAATCCCCAGGAGCTGTTGATCC	TAGTCCATACAACTATTGCTA
D	92UG024	sense	TAAACACAGTGGGGGGACATCAAG	GCACCCACCAAGGCAAAGAGAAGAGTGG	ATATCAAGCAGGACATAACAAGG
		antisense	AATCTGGGTTCGCATTTTGGACCA	CAACCCCAAATCCCCAGGAGCTGTTGATCC	CTATGATTACTATGGACCACACA
		sense[a]			GGAGCCAGTAGATCCTAACC
		antisense[a]			TTCTTCGTCGCTGTCTCC
AE	94TH304	sense	TAAACACAGTGGGGGGACATCAAG	ACCTGGAGGAGGAAATATAAAGGAC	AACTGTTAGAGGAGCTTAAA
		antisense	AATCTGGGTTCGCATTTTGGACCA	TTCCACAGCCAGGACTCTTGCTTG	CTATAGTCCACACTACTATTGCT
		sense[a]			AGATCCTAACCTAGAGCCCT
		antisense[a]			TATTGCTAAGATTAGCGCTACTA

[a] alternative primer combination used for the amplification of proviral sequences in cultures that did not demonstrate breakthrough virus replication.

Table S2. Mismatches between the gRNAs and viral target sequences

virus		gRNA [a]			
subtype	isolate		mismatches [b]	CFD [c]	RCE [d]
A	92UG029	gGag1	1	0.81	1.37
		gEnv2	1	0.60	0.54
		gTatRev	1	0.91	1.02
C	PHD79B8	gGag1	2	0.86 x 0.81 = 0.70	1.54 x 1.37 = 2.11
		gEnv2	0		
		gTatRev	0		
D	92UG024	gGag1	0		
		gEnv2	1*	0.67	0.12
		gTatRev	0		
AE	94TH304	gGag1	1	0.93	0.71
		gEnv2	0		
		gTatRev	2	0.91 x 0.86 = 0.78	1.02 x nd

[a] The color indicates the effect of the gRNA on virus replication (Figure 2): orange, no inhibition; yellow, delayed replication.

[b] Number of mismatching nucleotides between gRNA and viral target sequence. *, mismatch at Cas9 cleavage site.

[c] Cutting frequency determination (CFD) score based on the activity of single-nt mismatching gRNAs, as described by Doench et al. [29](values extracted from Figure 5e and Table S19 in this reference). If there are two mismatches, individual CFD values are multiplied together.

[d] Relative cleavage efficiency (RCE) based on the activity of single-nt mismatching gRNAs targeting 15 EMX1 sites, as described by Hsu et al. [30](values extracted from Figure 2 and Table S5 in this reference). If there are two mismatches, individual RCE values are multiplied together. nd, not determined.
